# Generative AI in Higher Education: Balancing Innovation and Integrity

**DOI:** 10.3389/bjbs.2024.14048

**Published:** 2025-01-09

**Authors:** Nigel J. Francis, Sue Jones, David P. Smith

**Affiliations:** ^1^ School of Biosciences, Cardiff University, Cardiff, United Kingdom; ^2^ Education, Institute of Biomedical Science (IBMS), London, United Kingdom; ^3^ Department of Biosciences and Chemistry, Sheffield Hallam University, Sheffield, United Kingdom

**Keywords:** generative artificial intelligence (GenAI), personalised learning, assessment practices, academic integrity, ethical frameworks

## Abstract

Generative Artificial Intelligence (GenAI) is rapidly transforming the landscape of higher education, offering novel opportunities for personalised learning and innovative assessment methods. This paper explores the dual-edged nature of GenAI’s integration into educational practices, focusing on both its potential to enhance student engagement and learning outcomes and the significant challenges it poses to academic integrity and equity. Through a comprehensive review of current literature, we examine the implications of GenAI on assessment practices, highlighting the need for robust ethical frameworks to guide its use. Our analysis is framed within pedagogical theories, including social constructivism and competency-based learning, highlighting the importance of balancing human expertise and AI capabilities. We also address broader ethical concerns associated with GenAI, such as the risks of bias, the digital divide, and the environmental impact of AI technologies. This paper argues that while GenAI can provide substantial benefits in terms of automation and efficiency, its integration must be managed with care to avoid undermining the authenticity of student work and exacerbating existing inequalities. Finally, we propose a set of recommendations for educational institutions, including developing GenAI literacy programmes, revising assessment designs to incorporate critical thinking and creativity, and establishing transparent policies that ensure fairness and accountability in GenAI use. By fostering a responsible approach to GenAI, higher education can harness its potential while safeguarding the core values of academic integrity and inclusive education.

## Introduction

Generative Artificial Intelligence (GenAI) has rapidly become a transformative factor in higher education, influencing assessment practices and pedagogical approaches to learning and teaching, offer unprecedented capabilities for personalised learning journeys and streamline assessment processes [1, 2]. When considering GenAI as an enabler, it can deliver substantial efficiency gains and enhanced educational outcomes through personalised learning pathways. The disruptive nature of GenAI as a technology and its emergence in higher education has raised a range of concerns around academic integrity, the authenticity of student work, and the potential reduction in students’ development of essential cognitive and creative skills [3, 4]. There is, then, a requirement for robust frameworks to manage the impact of GenAI responsibly [5, 6].

Ensuring that GenAI complements, rather than replaces, human proficiencies and insight is vital [1, 7]. A dual approach of utilising clear frameworks alongside reviewing and updating assessment practices can harness GenAI’s capabilities, whilst preserving the integrity of the educational experience. This paper summarises current research on GenAI’s impact on higher education assessment practices, exploring its opportunities and challenges as a tool for teaching. We provide a comprehensive overview of the current landscape and offer guidance for the responsible integration of GenAI in learning and assessment.

## Background

The field of Artificial Intelligence (AI) is a broad area of computer science that is focused on creating systems that can perform tasks typically requiring human intelligence. AI has evolved significantly over the past decade, driven by advancements in machine learning, natural language processing, and neural network architectures. Machine learning (ML) is a subset of AI that specifically deals with algorithms and statistical models that enable computers to learn from and make predictions or decisions based on data. ML is used in a range of applications, from finance [8] to biomedical science [9]. Here, we are considering GenAI, which is a form of ML trained on large datasets and capable of “generating” text, images, videos, or other data using generative models, often in response to prompts that were not part of its original training data set. Technologies such as GTP4 exemplify this form of AI and can produce coherent and contextually relevant text [1, 2]. Similarly, technology such as DALL-E has provided breakthroughs in creating high-quality images from textual descriptions, showcasing the versatility and potential of GenAI [10]. The progression from simple AI models to sophisticated LLMs has been driven by improvements in data processing capabilities, algorithmic efficiency, and the availability of vast training data. These developments have enabled GenAI systems to start performing complex tasks that were previously unattainable, thus opening new avenues for their application in all aspects of education [7].

## Ethical and Responsible Use of GenAI

GenAI has demonstrated that it can create realistic human-like text, believable images, videos, and other media. Similarly, many assessment approaches in HE are based on the ability to create novel content (the assessment) based on a prompt (the assessment brief) built on a foundation of prior knowledge (the training dataset). The rise of AI-generated content clearly poses challenges to maintaining academic integrity in this context. Richardson and Clesham [3] highlighted the difficulties in detecting AI-generated submissions, which can undermine the authenticity of student work. Such concerns regarding academic integrity in the context of GenAI are supported by more recent data highlighting challenges in distinguishing between student-generated and AI-generated work [11]. There are also significant concerns that GenAI detectors disproportionately disadvantage neurodiverse and non-native English language speakers [12], (See also article by Newton and Jones in this issue [13]). Therefore, a wide range of issues need to be acknowledged before considering the effective use of GenAI in an educational setting.

### Uncertainty

When presenting information, humans will often qualify their response with modifiers, such as “I will need to check that,” “As far as I understand,” or “To the best of my knowledge.” GenAI models tend to provide answers without equivocation in a manner that is confident and believable. However, due to the stochastic nature in which the outputs are generated, they can be incorrect or misleading, resulting in the spread of disinformation. There is also the danger of cognitive offloading, where the “thinking” is outsourced to the GenAI, which could lead to poor understanding by the student.

### Explainability

GenAI’s key challenge in identifying a “truth” is that it does not have a clear information source. LLMs like GTP are trained to construct sentences by making a series of guesses on the statistically likely “token”, or sequence of characters, that comes next. There is also an issue with using copyrighted information and open-access information without attribution. This lack of transparency around the source of the information provided would not pass the normal, stringent expectations of academic rigour.

### Bias

When training on a large corpus of text or image data, the LLMs naturally replicate any representative biases in its source. Given the data sets are inherently Western-centric and predominantly white male-focused, the overuse of GenAI can undermine recent work around inclusive practices and decolonisation of the curriculum. Addressing biases and ensuring fairness is paramount, as highlighted by Challen, Denny [14], and Lee, Resnick [15].

The Inclusive Higher Education framework proposed by Hubbard and Gawthorpe [16], emphasises the importance of creating inclusive and equitable educational environments by addressing systemic biases and promoting diversity; by extension, these principles can be applied to using GenAI. When considering the ethical and responsible use of GenAI, it is important to be aware of potential biases in the training data that could reflect and perpetuate existing inequalities, such as the underrepresentation of contributions by scientists from historically marginalised groups. Ensuring that GenAI technologies are deployed in a way that aligns with the framework’s commitment to equity and inclusion is crucial to avoid reinforcing these systemic issues.

### Environmental Impact

GenAI can have significant environmental costs. The training process alone, for example, is estimated to have emitted 284 tonnes of CO_2_ [17]. By 2040, the emissions from the Information and Communications Technology (ICT) industry are expected to reach 14% of the global emissions, with the majority of those emissions coming from the ICT infrastructure, particularly data centres and communication networks [18].

### Data Integrity

Many LLMs do not pass General Data Protection Regulation (GDPR) guidelines, which set strict regulations regarding the collection, storage, and processing of personal data. These guidelines also grant individuals certain rights, such as the right to access their data, the right to erasure, and the right to be informed about how their data is being used [19]. In the context of academia, this would mean that uploading student assessments to generate feedback or qualitative and quantitative research data to an LLM for analysis could breach local data handling guidelines.

### Digital Divide

Effective use of GenAI can enhance students’ understanding and learning. However, many of the more powerful LLMs and GenAI tools operate a subscription model for access, meaning there is an inherent monetary cost to use them. Access to the internet is already unevenly distributed. 2023 Pew Research data show that 83% of White adults have access to broadband internet, while only 68% of Black adults have similar access [20]. These existing barriers raise concerns around equity, with those having the means to access the more advanced tools and models being at an advantage over those who cannot, potentially widening the already problematic awarding gaps between white and global majority students. Hubbard [21] outlines that existing metrics can be reductive and fail to capture the full complexity of equity, particularly in educational outcomes; GenAI can potentially exacerbate this divide further.

Given these implications, the ethical use of GenAI in education requires frameworks that address fairness, transparency, and accountability. Such models advocate for the development and implementation of GenAI systems that are unbiased and ethically robust, ensuring that their integration into educational practices does not compromise equity or integrity or reinforce existing and embedded inequities [3]. An initiative driving this transparency of use is the AI Ethics Guidelines proposed by the Institute of Electrical and Electronics Engineers (IEEE), which advocate for developing transparent and accountable AI systems for the populations they serve [22].

GenAI in education raises several ethical concerns; systems must be designed and implemented to avoid perpetuating existing biases, such as those outlined above, and promote equitable educational opportunities. Additionally, AI-generated content can lead to false accusations of academic dishonesty. Mitigating these risks involves developing clear guidelines for GenAI use in assessments [23] (see Institutional Strategies and Governance below). Adhering to these pedagogical and ethical considerations allows educational institutions to maximise the benefits of GenAI while safeguarding the integrity and fairness of their educational practices.

## Theoretical Frameworks and Models

Understanding the impact of GenAI on education requires a multi-faceted theoretical approach [24]. GenAI tools can significantly improve educational outcomes by providing personalised feedback, facilitating language learning, and supporting both qualitative and quantitative research methodologies. There is capacity to increase learner engagement and motivation on the basis of robust ethical guidance and oversight on issues such as privacy, bias, and accuracy. Effective integration of GenAI tools into learning requires strategic planning and adherence to pedagogical principles. Here, we briefly explore several existing frameworks to contextualise GenAI’s role within educational settings, focusing on the interplay between pedagogical theory and the technological potential of GenAI [24].

### Social Constructivism

Constructivism proposes that learning is an active process where learners build their understanding of the world through experiences and interactions with the environment. A social constructivism framework emphasises learning as a collaborative, interactive process where knowledge is constructed within a social context, for example, interactive workshops or group activities [25]. GenAI can facilitate collaborative learning by providing tools that enhance communication and idea sharing among students, regardless of their linguistic or cultural backgrounds. This aligns with the principles of social constructivism, which posits that knowledge is constructed through social interactions and shared experience. GenAI tools can facilitate this approach by providing interactive environments and scaffolding student learning [26]. For instance, AI-powered discussion platforms, first used during the COVID-19 pandemic (e.g., Packback), can simulate peer interactions for remote learners, promoting engagement and a deeper understanding of course material [27, 28]. This reflects Vygotsky’s theory on the Zone of Proximal Development, which highlights the role of social interaction in learning processes [29].

### Personalised Learning

Personalised learning in the context of GenAI refers to the use of AI-driven tools and systems that tailor educational experiences to each student’s individual needs, preferences, and learning pace. At its core, personalised learning involves moving away from the traditional “one-size-fits-all” model of education. Instead, it focuses on understanding and addressing the unique requirements of each learner. These models advocate for customising educational experiences to meet individual learner needs, abilities, and interests [30, 31]. Popenici and Kerr [26] and Bennett [32] suggest various strategies to integrate GenAI into personalised learning experiences, including AI-powered teaching assistants to support student learning during authentic assessments that promote experiential learning assessments. GenAI can dynamically adjust content delivery and assessment methods based on continuous data analysis, modifying learning activities in real-time [26, 32]. This ensures that each student receives tailored support while learning, thus enhancing engagement and educational outcomes by aligning with individual learning trajectories and an appropriate level of academic challenge [2, 4, 33]. GenAI tools have also been demonstrated to effectively create assessments for Massive Open Online Courses (MOOCs), benefiting educators and learners alike. Students can employ the same methods to generate a variety of assessments for self-evaluating their progress in their studies [34].

### Competency-Based Learning

Competency-based learning assesses students’ ability to apply concepts in real-world scenarios, rather than through traditional memory-based assessments [35]. Behaviourist principles, particularly those related to reinforcement and feedback, are central to competency-based education. In this framework, learning is seen as a change in behaviour resulting from the acquisition of knowledge or skills, and students are assessed based on observable outcomes or competencies. Competency-based education also draws from constructivism by emphasising the learner’s active role in their education, allowing them to progress at their own pace as they build and demonstrate competencies through practical application. This type of learning activity may be delivered through case studies or data analysis and interpretation activities. The learning can be effectively assessed using laboratory-based competency tests, direct observations of practice (DOPs) or OSPEs (Objective Structured Practical Exams). GenAI can facilitate competency-based learning by providing detailed feedback on performance and identifying areas for improvement, thereby aiding learners in mastering specific competencies [4]. For instance, many MOOC platforms were developed with incorporated AI to offer personalised quizzes and real-time feedback, helping learners focus on areas that need improvement, which is crucial for competency development [36]. The focus is on fostering critical thinking, creativity, and problem-solving skills rather than simply recalling information [37]. When considering these theoretical frameworks together, it is apparent that GenAI’s impact on education and learning is complex and requires changes to more established practices. A holistic approach to the integration of GenAI can be achieved, that considers both the technological potential and the ethical implications of use. Such an approach will ensure that GenAI enhances rather than detracts from the educational experience.

## Impact on Assessment Practices

Moorhouse, Yeo [38] conducted a review of publicly available assessment guidelines from the top 50 universities in the Times Higher Education World University Rankings 2023. The focus of the review was on the content and advice provided for instructors regarding the use of GenAI in assessments. The review summarised that the redesign of coursework assessments to incorporate GenAI tools effectively was encouraged. This redesign involves designing assessment tasks with the intentional use of GenAI, emphasising critical thinking and creativity, focusing on the learning process over final outputs, and supporting staged assessments that allow for feedback and development. Alternatively, using in-class, closed-book assessments to prevent potential misuse of GenAI in producing answers and testing each individual student’s knowledge and understanding could be implemented. These assessments can remain authentic, such as case studies, data analysis, and evaluation of published articles or viva voce, as robust methods to assess critical thinking, creativity, and problem-solving skills.

### Re-Thinking Assessment Methods

AI’s capabilities necessitate innovative approaches to assessment design. Bobula [7] and Chan and Colloton [10] discuss the importance of creating assessments that require critical thinking and deeper understanding, which are currently more difficult for GenAI to replicate easily. Assessment methods include open-ended questions [39], project-based assessments [40], and working-world problem-solving tasks [41] that encourage original thought and application of knowledge, thereby reducing the potential for GenAI misuse [42]. A key shift in thinking about assessment design and the iterative creation of the piece of work to be submitted is needed. Project-based learning can address this need where students must engage in a process over time, such as research projects (capstone experiences), case studies, or design tasks, which require original thinking and continuous instructor feedback [40]. The development of assessments that allow students to use GenAI tools for certain parts of the task, followed by a critical evaluation of the AI’s output, is becoming more commonplace. Such approaches help students develop critical thinking skills about GenAI and its capabilities [43]. By using a product-orientated rather than a process-driven assessment model, it is more likely that even if students use GenAI to complete assessments, they will have to engage with, refine and update the outputs of tools and evidence ethical usage of the GenAI package selected [44]. Such process-orientated approaches reduces the likelihood of relying solely on GenAI to produce the final product. GenAI can also be directly incorporated into the assessment, for example, acting as examiners in assessments like Observed Structured Clinical Exams (OSCEs) addressing the historical concerns about human examiner reliability in this format [13]. Perkins et al. [45] set out a framework that provides clear guidelines for educators and students on acceptable AI usage at various assessment levels, from ideas generation through editing to evaluation of AI outputs to full integration as a “co-pilot.” The final stage of comprehensive integration utilises GenAI as a tool for collaboration and creativity, illustrating the diverse applications of these technologies beyond the realm of education. AI-generated content is increasingly used, for example, in the generation of code for data analysis, but it still requires human oversight and editorial interventions (REF). The partner review to this paper Jones and Newton (2024) detail a pragmatic guide for educators when designing assessments which takes into consideration GenAI use and its implications for learning [13].

### Supporting Autonomous Learning

GenAI has the capability to support and enhance self-regulated learning, leading to more autonomous learners. Self-regulated learning refers to the ability of students to plan, monitor, and reflect on their learning and problem-solving capabilities. The use of AI-powered chatbots to create questions for students to answer that are based on current levels of understanding and achievement within a course, to develop personalised study plans based on strengths, weaknesses and needs or to provide tutoring and feedback to students are all examples of how students can leverage GenAI to develop self-autonomy [46]. The use of personalised learning approaches leads to improved student engagement, better understanding of the material, and enhanced academic performance [26, 30–33].

Empirical studies have shown that GenAI is effective in delivering personalised learning and improving student autonomy and confidence. For example, a review by Gligorea, Cioca [47] found that GenAI-driven adaptive learning systems, which modify instructional content based on learner responses, significantly improve student performance. Crompton and Burke [48] and Kaledio, Robert [49] highlight that consistent student interactions with intelligent GenAI tutoring systems in university settings lead to improved overall academic achievement. This suggests that GenAI not only tailors the learning experience to individual needs but also enhances the effectiveness of educational interventions, supporting the integration of GenAI into frameworks in higher education designed to develop self-regulation and learner autonomy.

### Automation and Efficiency

The integration of GenAI in higher education has the potential to positively impact marking and feedback processes. Automated systems can offer instant, consistent, and unbiased feedback, allowing educators to focus on more intricate tasks. Research by Bearman and Luckin [1] and Hooda, *Rana* [2] demonstrates how GenAI can enhance the efficiency and reliability of assessments. Messer, Brown [50] extend this by showing that automated grading tools can enhance student satisfaction by providing near-instantaneous feedback and multiple resubmission opportunities [50]. For instance, GenAI tools can be used to assess written assignments, code, and the outputs of multiple-choice tests, providing immediate and detailed feedback to students.

The study by Balfour [51] demonstrates that automated essay scoring systems powered by GenAI expedite the grading process and maintain consistency and reliability in scoring across large cohorts. It has been shown that essays marked by humans and GenAI are internally consistent (i.e., human-human and AI-human scores) and that the mean differences between human-human and AI-human scores are not statistically significant [52]. Perera and Lankathilake [53] discuss how integrating multi-model GenAI tools can revolutionise education by providing detailed and timely feedback while addressing ethical considerations. An important consideration will be how the use of GenAI in this context is explained to students, including the benefits of timely and impartial feedback against the loss of interaction with academic staff, as well as maintaining data integrity and GDPR compliance.

By leveraging AI’s strengths while addressing its challenges, educational institutions can enhance the effectiveness and integrity of assessment practices, ensuring they meet the evolving needs of both educators and students.

### Perspectives and Attitudes

Surveys of educators and students reveal diverse attitudes towards GenAI in education [54]. The analysis indicates a general recognition of AI’s benefits in supplementing learning, assessment efficiency and feedback. However, there is also significant apprehension regarding the potential for academic dishonesty and its impact on learning processes.

### Educator Perspectives

Educators have reported varied preferences regarding GenAI being integrated into teaching practices [6]. The literature highlights that many educators appreciate GenAI’s potential for enhancing efficiency and streamlining processes [55]. Concerns around ethical and academic integrity from an assessment point of view are, however, also frequently cited. The debate centres on the balance between the benefits of GenAI in augmenting learning outcomes and the risks of undermining the authenticity of students’ work [56].

The use of GenAI in education, particularly in creative fields, has prompted a reconsideration of how creativity and creative outputs are assessed. This technology challenges traditional pedagogical practices and requires new approaches to foster creativity in the presence of GenAI tools [57]. Farrelly, Farrell [58] in their reflections on EdTech and GenAI observed that educators are facing numerous complexities and constraints in adapting these new digital tools into their practices and cite a need for GenAI literacy within the staff base [58]. Similar observations were made by Lacey and Smith [59], with educators feeling the need to keep up to date balanced against the time needed to do this and Walczak and Cellary [60] also highlighted the need for support in this new digital literacy.

To ensure that educators are well-equipped to navigate the evolving landscape of GenAI in education, it is essential that they become GenAI literate, developing a robust understanding of both the capabilities and limitations of GenAI technologies. The GenAI Readiness Framework proposed by Luckin, Cukurova [61], is a structured, step-by-step approach, offering a practical pathway for staff to achieve this literacy. By engaging in this process, educators can tailor GenAI tools to meet specific educational challenges, enhance their teaching practices, and critically assess the ethical implications of GenAI integration. This proactive approach empowers educators to harness GenAI effectively and ensures that AI’s adoption enhances rather than undermines the educational experience.

The authors propose a seven-step framework, “EThICAL”, to guide AI Readiness;

1. Excite: engage staff with AI possibilities.
2. Tailor and Hone: identify specific challenges that AI can address.
3. Identify: determine available data and its relevance to the identified challenges.
4. Collect: gather additional data needed to address these challenges.
5. Apply: select and implement appropriate AI techniques.
6. Learn: analyse the results and learn from the data.
7. Iterate: refine the process based on outcomes and repeat if necessary.

The authors acknowledge that the interconnectedness of educational systems means that changes in one area can have far-reaching impacts, which must be considered when integrating GenAI into existing teaching methodologies [61].

### Student Perspectives

Student reactions to GenAI in assessments are mixed. Chan and Hu [5] report that while some students value the personalised feedback and learning support GenAI can provide, others are concerned about the impact on creativity and the authenticity of their work. These concerns underscore the importance of transparent institutional policies and guidelines and the need to educate students about ethical GenAI use [62]. While students generally trust GenAI for certain tasks like grammar checking, they prefer human educators for feedback on assessments [63].

Comparing attitudes across different assessment scenarios shows a need for tailored approaches to GenAI integration that address specific concerns and contexts. A recent report by Jisc [64] highlights that students are already using GenAI tools for a range of tasks, including content creation, programming and personal support, often viewing these tools as digital assistants that enhance their creativity and productivity. However, they express concerns about over-reliance on AI, the potential loss of individual creativity, and the impact on their intellectual development. Additionally, issues of equity, bias, and the ethical use of GenAI in assessments are prominent, with students advocating for clearer institutional guidelines to ensure fair and responsible GenAI integration. Recommendations include developing robust guidelines for GenAI use and ensuring that GenAI complements rather than replaces traditional teaching methods alongside the need for responsible and ethical implementation [53]. By understanding and addressing these varied perspectives, educational institutions can better navigate the challenges and opportunities presented by GenAI in assessment practices.

## Institutional Strategies and Governance

### Policy Development

Developing clear guidelines for the responsible adoption of GenAI in higher education is crucial and has been mentioned throughout this review. Many institutions are developing a range of policy responses to manage the impact of AI, however progress is slow. Moorhouse, Yeo [38] found that less than half of the reviewed universities have developed publicly accessible guidelines addressing GenAI use. While Barrett and Pack [54] showed that over 94% of educators responding to a survey reported their institution as not having a clear GenAI policy, and nearly 90% had never provided training to students on the acceptable usage of GenAI.

GenAI, specifically its ability to learn and improve, poses a conceptual challenge, at some point, the rate of change in the technology will be faster than the rate at which regulation can be introduced and applied to that new technology. Ifenthaler, Majumdar [65] advocate for proactive policies that anticipate the challenges posed by rapid technological changes rather than reactive measures that may be overly restrictive. This includes creating frameworks that align educational data use with broader evidence-based practices; hence, policy needs to be responsive, flexible, adaptable and based on principles that can remain the same while technology changes.

Existing policies vary widely, with some institutions enthusiastically adopting GenAI technologies and integrating GenAI literacy into their curricula. In contrast, others are more cautious, focusing on the potential risks and ethical dilemmas. Farrelly, Farrell [58], Chan [66], Perera and Lankathilake [53] and Ifenthaler, Majumdar [65] set out guidance covering areas such as academic integrity, assessment design, and student communication. There is also a growing focus on responsible GenAI use, with policies emphasising transparency, accountability, inclusiveness, and fair use of GenAI technologies. Common areas include ethical considerations, responsible use, and the need for educators to act as “moral exemplars,” guiding students in navigating the complex ethical landscape surrounding AI [65]. Impact on teaching and learning through the adoption of teaching methods assessment strategies, addressing ethical challenges, and fostering collaboration among stakeholders [53], and addressing academic misconduct through clear guidelines, approaches for detection and prevention, and promoting academic integrity in AI use [66].

The Royal Melbourne Institute of Technology (RMIT) University created referencing guidelines for AI-generated content, ensuring academic integrity and promoting the use of these tools in assessment tasks [67]. Jisc, the UK digital, data, and technology agency focused on tertiary education, research, and innovation, has provided links to institutional policy and toolkits on GenAI use [68] and a common theme of allowing GenAI in assessments but giving clear acknowledgement of how the tools were used.

### Training and Support

Digital competency is the ability to effectively use digital technologies, tools, and platforms to accomplish tasks, solve problems, and communicate in various contexts. This competency covers a broad spectrum of skills, from basic computer literacy to more advanced capabilities such as coding, data analysis, digital content creation, cybersecurity, and ethical use of digital information. In the context of GenAI, digital competency extends further to include the skills needed to interact with and leverage GenAI tools that create new content. This involves not just understanding how to use GenAI tools but also the ability to craft precise prompts to guide GenAI systems in producing the desired outputs. Such GenAI literacy is essential for both educators and students and has been highlighted as a significant concern for both academics and students. Richardson and Clesham [3] emphasise the importance of training programs that equip users with the necessary skills to utilise GenAI tools effectively and responsibly. In their review, Korzynski, Mazurek [69] explore GenAI prompt engineering as a digital competency and argue that this is a key graduate skill and present a conceptual framework that encompasses different strategies for GenAI prompt engineering. Cain [70] sets out how training in prompting helps students and educators transition from passive recipients to active co-creators of their learning experiences. For instance, Webb, Fluck [71] demonstrated the effectiveness of targeted training sessions in enhancing confidence and competence in integrating GenAI tools into curricula. Institutions, government bodies and learned societies can and do offer workshops, online courses, and resource centres dedicated to GenAI education, ensuring all stakeholders are prepared to engage with GenAI technologies in an informed manner.

The need for comprehensive GenAI literacy programs for both staff and students is crucial as institutions seek to incorporate GenAI into educational practices. Cultivating GenAI literacy among academic colleagues and students fosters a better understanding of both the capabilities and limitations of GenAI. By implementing these strategies, education institutions can ensure the ethical and effective integration of AI, creating an environment that encourages innovation while upholding academic integrity and fairness.

## Future Directions and Research Needs

Several systematic reviews have been conducted into the use of GenAI in higher education. These studies suggest current gaps in GenAI in education research include addressing ethical and privacy concerns, integrating GenAI with educational theories and practices, and exploring fairness, accountability, and transparency [72–74]. Ali, Murray [75] also highlight several gaps including a lack of comprehensive understanding of GenAI’s long-term effects on learning outcomes and a need for more studies on the effectiveness of GenAI tools across diverse educational settings and disciplines.

Further developments in the field then will require: Longitudinal Studies: Investigating the long-term impact of GenAI integration on student performance and learning processes; Cross-Disciplinary Analysis: Assessing the effectiveness and challenges of GenAI tools in various disciplines, Ethical Frameworks: That develop robust guidelines for GenAI use in education, ensuring fairness and equity, AI Literacy: Evaluating the effectiveness of GenAI literacy programs for educators and students, Policy Impact: Studying the effectiveness of institutional policies and frameworks in managing GenAI integration in higher education. Addressing these gaps will help develop a deeper understanding of GenAI’s role in education and guide the responsible and effective use of these technologies.

## Conclusion

This review highlights the transformative impact of GenAI on learning and teaching in HE, including enhanced automation, personalised learning, and innovative assessment methods. It also identifies significant challenges, such as maintaining academic integrity and addressing ethical concerns in appropriate use of AI. HEIs need to develop comprehensive and consistent policies for GenAI integration, emphasising their ethical use and fairness. Training programs for GenAI literacy among educators and students will be crucial for effective implementation. Institutions have an obligation to design robust frameworks to ensure GenAI tools complement traditional educational methods and enhance student learning experiences. GenAI holds great promise for transforming HE, but its integration must be managed carefully to preserve academic integrity and promote equitable learning opportunities. Ongoing research and collaboration among educators, policymakers, and GenAI developers will be essential in harnessing AI’s full potential while mitigating its risks.

## Abbreviations

AI, artificial intelligence; GenAI, generative artificial intelligence; LLMs, large language models; MOOC, Massive Open Online Course.
